# Albumin Clearance as a Novel Predictor of Relapse in Adults with Minimal Change Nephrotic Syndrome

**DOI:** 10.34067/KID.0000000000000169

**Published:** 2023-06-29

**Authors:** Ryohei Yamamoto, Yoshitaka Isaka

**Affiliations:** 1 Health and Counseling Center, Osaka University, Toyonaka, Japan; 2 Laboratory of Behavioral Health Promotion, Department of Health Promotion, Graduate School of Medicine, Osaka University, Toyonaka, Japan; 3 Department of Nephrology, Graduate School of Medicine, Osaka University, Suita, Japan

**Keywords:** nephrotic syndrome

Minimal change nephrotic syndrome (MCNS) is one of the major primary glomerular diseases in adults with nephrotic syndrome, along with membranous nephropathy and FSGS. Compared with membranous nephropathy and FSGS, MCNS is characterized by steroid-sensitive nephrotic syndrome with high selectivity for proteinuria. Approximately 80% patients achieved remission of proteinuria within 2 months of immunosuppressive therapy.^1^ Major predictors of early remission of proteinuria in adult patients with MCNS are young age,^1–3^ low albumin concentration,^1,3,4^ and normal kidney function,^2,4^ along with no acute kidney injury.^1,3^ Proteinuria selectivity index (SI), defined by Cameron and Blandford as a clearance ratio of immunoglobulin G (IgG; 150 kDa) to transferrin (80 kDa), is widely used to measure the glomerular permeability for macromolecules. Although MCNS is characterized by a lower SI (≤0.2) than other glomerular diseases,^5^ the clinical relevance of SI with the incidence of remission in adult patients with MCNS remains to be elucidated.^6^

Despite steroid sensitivity, adult patients with MCNS frequently relapse, requiring long-term immunosuppressive therapy. Among adult patients with steroid-sensitive MCNS, more than 40%–50% of patients relapsed after achieving remission of proteinuria.^1,2,4^ Thus, the treatment goals in adult patients with MCNS are prevention of frequent relapses of proteinuria and suppression of adverse effects of long-term immunosuppressive therapy. Identifying patients at a high risk of relapse of proteinuria is crucial for these goals. However, contrary to the major predictors of remission of proteinuria, few clinical factors have been identified as major predictors of relapse of proteinuria in adult patients with MCNS, except for young age.^2^

In this issue of *Kindey360*, Kuno and colleagues identified albumin clearance (C_ALB_) as a novel predictor of relapse of proteinuria in adult patients with steroid-sensitive MCNS.^7^ This retrospective cohort study included 103 adult patients with nephrotic syndrome who were diagnosed with MCNS using kidney biopsy between 2010 and 2020 in four hospitals in Japan and achieved remission of proteinuria after initiating corticosteroid therapy. Because urinary albumin excretion was not measured in this retrospective cohort study, the authors estimated the baseline C_ALB_ (eC_ALB_) at kidney biopsy using the equations to estimate urine albumin-to-creatinine ratio from protein-to-creatinine ratio in male and female patients with proteinuria.^8^ Mean eC_ALB_ of 103 patients was 2.27 *μ*l/min (interquartile range, 1.33–4.01). Patients with higher eC_ALB_ were likely to have lower serum albumin levels, higher urinary protein excretion (UPE), and higher SI than those with lower eC_ALB_. During the 2-year observational period after the remission of proteinuria, relapse of proteinuria was observed in 44 patients (42.7%). Receiver-operating characteristic curve analyses showed that eC_ALB_ had the highest value of area under the curve, followed by serum albumin level, total protein clearance level, UPE, and SI, suggesting that eC_ALB_ predicted relapse of proteinuria more accurately than these factors. Besides the time from initiation of corticosteroid therapy to remission of proteinuria, eC_ALB_ was identified as a significant predictor of relapse of proteinuria in a Cox proportional hazard model adjusting for age, estimated glomerular filtration rate, UPE, and time to remission of proteinuria (per 1.0 *μ*l/min, adjusted hazard ratio 5.027 [95% confidence interval, 1.88 to 13.47]). The authors elaborately showed that doses of prednisolone 1, 2, 3, 6, 12, 18, and 24 months after initiating immunosuppressive therapy were clinically comparable among patients with baseline eC_ALB_ quantiles, suggesting that the results of this study were hardly confounded by immunosuppressive therapy during the observational period. The authors concluded that eC_ALB_ was a clinically useful predictor of relapse of MCNS, which is easily calculated from serum albumin level and UPE.

In addition to confirming that time to remission was associated with the incidence of relapse of proteinuria,^9^ this study identified eC_ALB_ as a novel predictor of relapse of proteinuria in adult patients with steroid-sensitive MCNS. Because albumin is a negatively charged molecule with a low molecular weight (66 kDa), the authors assumed that C_ALB_ might reflect glomerular hyperpermeability with impairment of glomerular charge selectivity. Given that patients with higher C_ALB_ had more severe impairment of the glomerular charge barrier and, therefore, were more vulnerable to relapse of proteinuria, those with higher C_ALB_ might be less likely to achieve remission of proteinuria, which was not assessed in this study. This study should have clarified the association between C_ALB_ and remission of proteinuria, in addition to relapse of proteinuria.

The underlying mechanism of the association between C_ALB_ and the incidence of relapse of proteinuria is unknown. Conventionally, albuminuria is mainly due to glomerular hyperpermeability with impairment of the glomerular charge and size barrier. On the basis of the conventional glomerular-centric model, C_ALB_ is an index of glomerular hyperfiltration of albumin, a major small serum molecule. Recent studies have suggested that glomerular hyperpermeability of albumin is primarily a size-selective event, and albuminuria is mainly ascribed to impaired uptake of albumin in the proximal tubule rather than glomerular hyperfiltration of albumin.^10^ Albumin is normally filtered in the glomerular barrier to a great extent. Most of the filtered albumin is retrieved in proximal tubule cells and returned to the blood supply. Suppression of albumin retrieval in the proximal tubule may lead to albuminuria. On the basis of this nephron model, C_ALB_ is an index of albumin uptake in the proximal tubules. Before clarifying the role of C_ALB_ in the relapse of proteinuria, albumin kinetics should be investigated in more detail.

In conclusion, this retrospective cohort study showed that eC_ALB_ was a novel predictor of relapse of proteinuria in adult patients with MCNS, which is easily calculated from serum albumin concentration and urinary protein-to-creatinine ratio. The results of this study may provide a clinically useful tool to stratify adult patients with MCNS at a high risk of relapse proteinuria, although they should be ascertained in well-designed cohort studies. In addition to the external validity of the findings of this study, albumin kinetics should be investigated in more detail to clarify the pathophysiological mechanism between C_ALB_ and relapse of proteinuria in MCNS.
